# Interface Catalysts of In Situ-Grown TiO_2_/MXenes for High-Faraday-Efficiency CO_2_ Reduction

**DOI:** 10.3390/molecules30194025

**Published:** 2025-10-09

**Authors:** Shaun Debow, Zichen Shen, Arjun Sathyan Kulathuvayal, Fuzhan Song, Tong Zhang, Haley Fisher, Jesse B. Brown, Yuqin Qian, Zhi-Chao Huang-Fu, Hui Wang, Zachary Zander, Mark S. Mirotznik, Robert L. Opila, Yanqing Su, Yi Rao

**Affiliations:** 1 U.S. Army Combat Capabilities Development Command Chemical Biological Center, Research & Operations Directorate, Aberdeen Proving Ground, Aberdeen, MD 21010, USAzachary.b.zander.civ@army.mil (Z.Z.); 2 Department of Chemistry and Biochemistry, Utah State University, Logan, UT 84322, USAzhichao.huangfu@usu.edu (Z.-C.H.-F.); hui.wang@usu.edu (H.W.); 3 School of Aerospace and Mechanical Engineering, University of Oklahoma, Norman, OK 73019, USAyanqing.su@ou.edu (Y.S.); 4 Department of Electrical and Computer Engineering, University of Delaware, Newark, DE 19711, USA; mirotzni@udel.edu; 5 Department of Materials Science and Engineering, University of Delaware, Newark, DE 19711, USA; opila@udel.edu

**Keywords:** TiO_2_/MXene electrode, CO_2_ reduction, interfacial catalysis, charge transfer, MXenes

## Abstract

Climate change and the global energy crisis have led to an increasing need for greenhouse gas remediation and clean energy sources. The electrochemical CO_2_ reduction reaction (CO_2_RR) is a promising solution for both issues as it harvests waste CO_2_ and chemically reduces it to more useful forms. However, the high overpotential required for the reaction makes it electrochemically unfavorable. Here, we fabricate a novel electrode composed of TiO_2_ nanoparticles grown in situ on MXene charge acceptor 2D sheets with excellent CO_2_RR characteristics. A straightforward solvothermal method was used to grow the nanoparticles on the Ti_3_C_2_T*_x_* MXene flakes. The electrochemical performance of the TiO_2_/MXene electrodes was analyzed. The Faradaic efficiencies of the TiO_2_/MXene electrodes were determined, with a value of 99.41% at −1.9 V (vs. Ag/AgCl). Density functional theory mechanistic analysis was used to reveal the most likely mechanism resulting in the production of one CO molecule along with a carbonate anion through ∗CO, ∗O, and activated CO_2_^2−^ intermediates. Bader charge analysis corroborated this pathway, showing that CO_2_ gains a greater negative charge when TiO_2_/MXene serves as a catalyst compared to MXene or TiO_2_ alone. These results show that TiO_2_/MXene nanocomposite electrodes may be very useful in the conversion of CO_2_ while still being efficient in both time and cost.

## 1. Introduction

Climate change and the global energy crisis have led researchers to turn to CO_2_ reduction as a means of greenhouse gas remediation to produce a clean energy source. The process of harvesting waste CO_2_ and chemically reducing it to a more useful form promises to address both issues but is energy-intensive. For this reason, efficient photo-, electro- and photoelectro-catalytic processes to promote the CO_2_ reduction reaction (CO_2_RR) are of great interest. The use of TiO_2_ as a catalyst is broad and multifaceted, which has led to many reviews on the process [1,2,3,4,5,6,7,8,9,10,11]. Of particular interest are the catalytic processes occurring at the TiO_2_ surfaces [5,10,11]. Over 50 years ago, TiO_2_ was demonstrated as a catalyst in water splitting [12]. By nature, the surface characteristics of a heterogeneous catalyst play a crucial role in its catalytic abilities. To this end, TiO_2_ surface structures and properties have been studied extensively with high-resolution scanning tunneling microscopy (STM) [13,14], scanning electron microscopy (SEM) [15,16], and transmission electron microscopy (TEM) [17,18]. TiO_2_ has found a role as a catalyst in the reduction of CO_2_ as well [19]. However, the catalytic reduction of CO_2_ to form methane or methanol on TiO_2_ nanoparticles is not without flaws: the close proximity of the reduction and oxidation half-reactions can lead to unwanted carrier recombination, and the reaction pathways proposed for the catalysis are complex and still debated [20,21,22]. As a potential solution, cocatalysts have been introduced to assist in the catalytic CO_2_RR by TiO_2_. Systems such as TiO_2_/Pt, MgO/Pt/TiO_2_, Ag/TiO_2_, and many more have been investigated for their catalytic potential in CO_2_RR and their variable performances have been cataloged [23]. One such cocatalyst, Ti_3_C_2_ (MXene), shows particular promise due to the high performance of the combined TiO_2_/Ti_3_C_2_ material and the absence of precious materials [24].

MXenes encompass a broad family of 2D layered materials composed of transition metal carbides and nitrides, with applications spanning from environmental remediation to photonics [25,26,27]. Specifically, Ti_3_C_2_T*_x_* (T=F, O, or OH) was first fabricated in 2011 by Naguib et al. by selectively etching away the Al layer in MAX phase materials (Ti_3_AlC_2_) [28,29]. Although carbide MXenes take the general form of Ti_n+1_C_n_T*_x_*, further references to MXene in this work refer specifically to Ti_3_C_2_T*_x_*. MXenes have found uses in flexible light-emitting diodes (LEDs) [30], hydrogel sensors [31], brain activity recording [32], and adjustable-focus ocular lens devices [33]. Of particular interest are the energy storage capabilities of MXenes and their electrochemical uses [34,35,36,37]. Such charge storage capabilities have been ascribed to the layered structure of MXenes, where it has been shown that Na^+^ ions are reversibly transported into and out of the interlayer space resulting in expansion and contraction, respectively, in aqueous electrolytes [38]. Such intercalation of cations by MXenes has also been demonstrated for other species, both spontaneously and electrochemically, where the 2D sheets become capacitors [39]. In fact, MXene-based solid-state supercapacitors have been successfully deposited onto conductive polymer threads, advancing the usefulness of the material to a flexible system [40]. Due to these properties, MXene is an excellent host material for TiO_2_ photocatalysts acting as a charge acceptor and scaffold.

TiO_2_/MXene composite materials were developed some time ago and boasted increased performance over the individual components [41,42,43,44,45]. The first demonstration of a TiO_2_/MXene catalyst was for the photocatalytic CO_2_RR, and was achieved by calcining Ti_3_C_2_ [24]. This work monitored the CO_2_RR to methane and concluded that the mechanism involved the MXene scaffold preventing fast charge recombination at the TiO_2_ surfaces and emphasized that the in situ growth of TiO_2_ on MXene was crucial in enhancing charge separation. In the same year, a solvothermal process for the fabrication of TiO_2_/MXene was published and analyzed for its photocatalytic potential [46], finding that TiO_2_/MXene readily degraded carbamazepine through ·OH and ·O_2_ species. Electrocatalytically, TiO_2_/MXene composites were tested for their ability to reduce N_2_ to NH_3_, showing superior efficiency under acidic conditions and excellent durability [47]. In an interesting approach, TiO_2_ was married to a hybrid Ti_3_C_2_/Ru cocatalyst which provided enhanced charge separation that promoted the hydrogen evolution reaction (HER) [48]. Increased catalytic activity of TiO_2_/MXene over TiO_2_ in the oxidative dehydrogenation of ethane has also been observed, where the defects in the composite surface relative to TiO_2_ were shown to contribute to a 4× increase in performance [49]. With specific regard to CO_2_ reduction, attempts at designing TiO_2_/MXene-based materials have been made including C_3_N_4_/Ti_3_C_2_T*_x_*/TiO_2_ [50,51], Ru-Ti_3_CN/TiO_2_ [52], TiO_2_/Ti_3_CN [53], and even tailoring the anatase/rutile moieties of TiO_2_ in TiO_2_A/Ti_3_C_2_/TiO_2_R MXene [54]. Due to the photocatalytic activity of TiO_2_, most of the work to date covering the catalytic CO_2_RR by TiO_2_/MXene has focused on the photocatalytic process. However, this has led to the direct electrocatalytic counterpart to be overlooked.

Here, we fabricate a novel electrode composed of TiO_2_ nanoparticles grown in situ on MXene charge acceptor 2D sheets with excellent electrocatalytic CO_2_RR characteristics. A straightforward solvothermal method was used to grow the nanoparticles on the Ti_3_C_2_T*_x_* MXene flakes for electrochemical CO_2_RR. After thorough characterization of the composite material, we used experimental and theoretical analyses to hypothesize a mechanism to explain the high Faradaic efficiency of the CO_2_RR.

## 2. Results and Discussion

**Figure 1:**
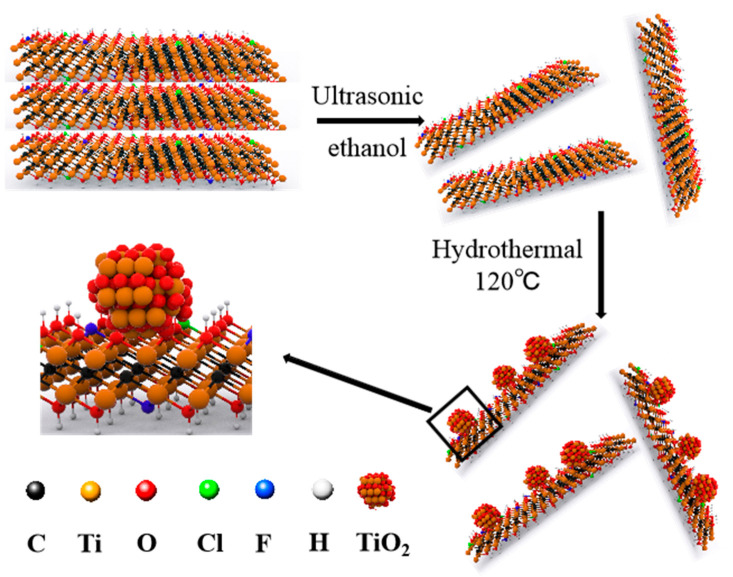
Schematic illustration of TiO_2_/MXene preparation procedure.

**Figure 2:**
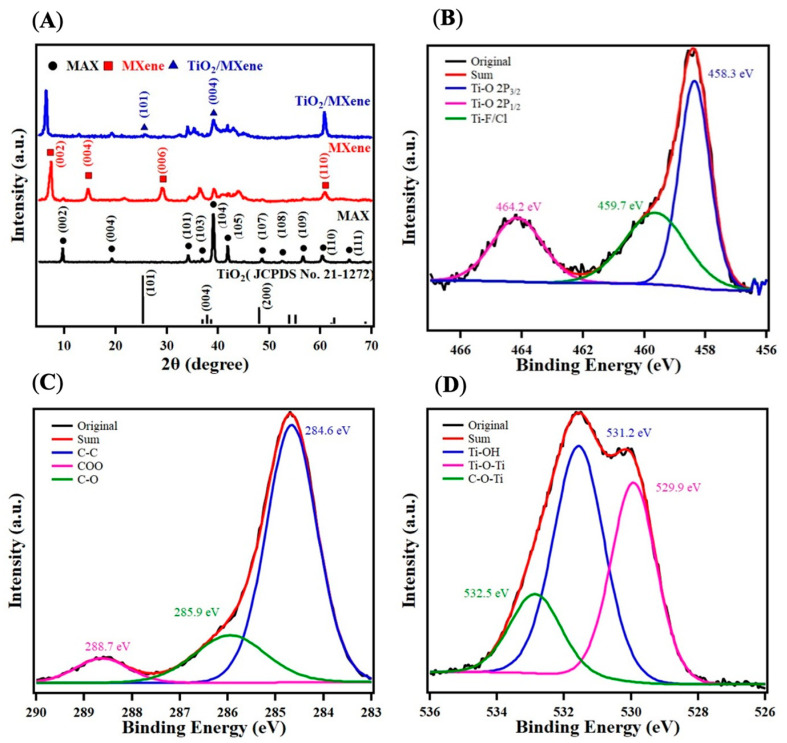
(**A**) XRD spectra of TiO_2_/MXene, MXene, MAX, and TiO_2_ (top to bottom). High-resolution XPS spectra of (**B**) Ti 2p, (**C**) C 1s, and (**D**) O 1s for TiO_2_/MXene.

TiO_2_/MXene catalysts were fabricated as described below and illustrated in Figure 1. To characterize our samples, the XRD scattering patterns of TiO_2_/MXene, MXene, and pristine MAX are presented in Figure 2A. After etching, a shift of the (002) peak from 9.64° to 7.28° and the elimination of most intense peaks at 39.06° indicated removal of Al layers in MAX and the formation of exfoliated MXene [55]. After hydrothermal processing, there were two new peaks at 25.76° and 39.14° in TiO_2_/MXene, which correspond to the (101) and (004) facets of anatase TiO_2_ [56]. Furthermore, the peak shift from 7.28° to 6.42° indicated an increase in the interlayer distance from the exfoliation of TiO_2_/MXene after the solvothermal treatment.

Next, we investigated the chemical state and composition of TiO_2_/MXene and pristine MXene by X-ray photoelectron spectroscopy (XPS). The presence of Ti, O, C, F, and Cl is shown in Figures 2B–D, S2 and S3. The F and Cl elements present on the surface of MXene are a product of HF etching. For the Ti 2p region, the peaks at 458.3, 459.7, and 464.2 eV correspond to the Ti–O 2p_3/2_, Ti–F/Cl, and Ti–O 2p_1/2_, respectively. The C 1s region was fitted with three peaks at 284.6, 285.9, and 288.7 eV, ascribed to the C-C, C-O, and O-C-O bonds, respectively [6]. The O 1s region spectra can also be divided into three subpeaks at 529.9, 531.2, and 532.5 eV, which are ascribed to oxygen in TiO_2_ (Ti–O–Ti), oxygen in the surface hydroxyl group (Ti–O–H), and C–O–Ti, respectively. Compared with MXene [57], all signals in TiO_2_/MXene displayed negative shifts, indicating an increased electron transfer from MXene to TiO_2_. As a result, a strong interfacial interaction can be achieved at the TiO_2_/MXene interface, which may enhance the performance toward electrocatalyzing the CO_2_ reduction.

**Figure 3:**
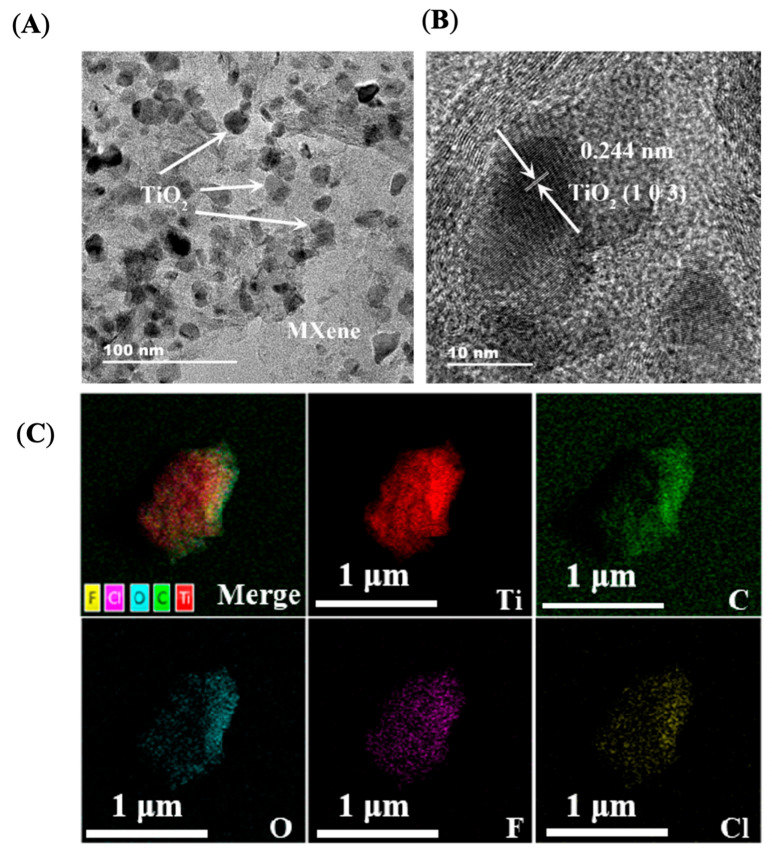
TEM (**A**) and HRTEM (**B**) images of TiO_2_/MXene nanocomposite. (**C**) Elemental mapping results of TiO_2_/MXene.

The morphology and microstructure of the synthesized TiO_2_/MXene were characterized by TEM and HRTEM. As shown in Figure 3A, the exfoliated MXene nanosheets showed single (or few)-layered and flake-like morphology using TEM. TiO_2_ nanoparticles with a size of 10–20 nm were also observed to be uniformly distributed on the surface of MXene nanosheets. As shown in Figure 3B, lattice fringes with a spacing of 0.244 nm were observed, which were ascribed to the (103) plane of anatase TiO_2_. Energy-dispersive X-ray (EDX) characterization was also conducted and the atomic ratio of Ti:O was found to be 2.96:1 (Figure S4). The elemental mapping results of the TiO_2_/MXene further confirm that the distribution of Ti and O elements of the in situ formation of TiO_2_ was monodisperse on the MXene surface, as shown in Figure 3C. TiO_2_ nanoparticles were grown in situ on MXene surfaces, endowing robustness to the microstructure and morphology.

**Figure 4:**
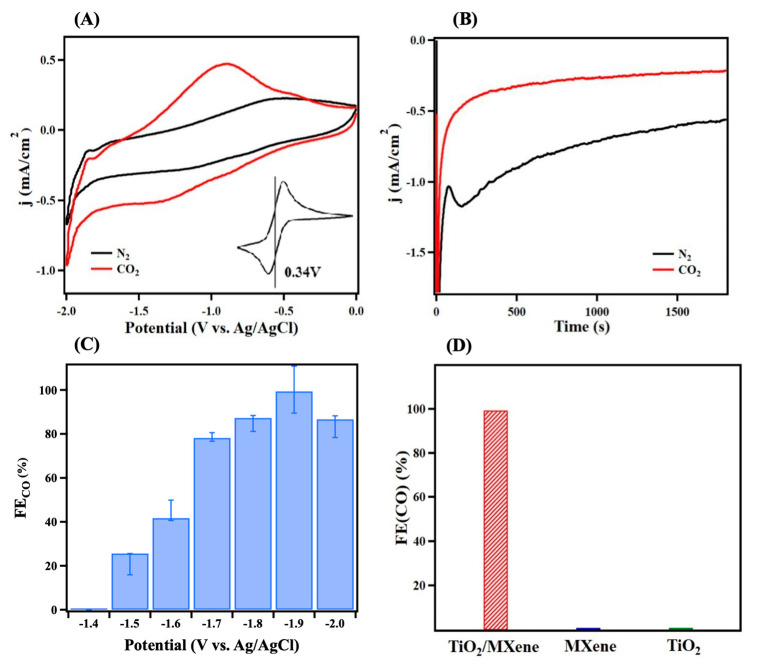
Electrocatalytic performance of reduction of CO_2_ to CO. (**A**) Cyclic voltammograms (CVs) of TiO_2_/MXene and MXene in electrolyte saturated with N_2_ (black) and CO_2_ (red). Inset shows reference E_0_ value for (Fc/Fc^+^) standard; (**B**) controlled potential colometry (CPC) at potential of −1.9 V in electrolyte saturated with N_2_ (black) or CO_2_ (red); Faradaic efficiencies (FEs) of (**C**) TiO_2_/MXene at different potentials and (**D**) TiO_2_/MXene, MXene, and TiO_2_ at −1.9 V.

The electrochemical conversion of CO_2_ to CO was investigated by cyclic voltammetry (CV) and controlled potential coulometry (CPC). Figure 4A shows the CVs collected using the TiO_2_/MXene electrocatalyst in the electrolyte saturated with N_2_ (black) or CO_2_ (red) gas. Here, we see a clear difference between the CVs upon introduction of CO_2_ during both increasing and decreasing potentials. In the N_2_-purged solution, TiO_2_/MXene showed a typical capacitive profile due to the CP support. The peak at −1.3 V is attributed to reduction from Ti^4+^ to Ti^3+^ on the TiO_2_ surface. With the CO_2_-saturated electrolyte, an apparent cathodic current was observed, corresponding to efficient CO_2_ conversion. Figure 4B shows the CPC scans of the N_2_-(black) or CO_2_-purged (red) electrolyte at a constant potential of −1.9 V. As such, the TiO_2_/MXene nanocomposites show significant potential-dependent CO_2_RR catalytic properties.

To investigate the performance of the TiO_2_/MXene catalyst during CO_2_RR, the Faradaic efficiency for CO production (FE_CO_) was calculated for various potentials. As shown in Figure 4C, it was observed that CO production started at potentials more negative than −1.4 V and peaked at 99.41 ± 0.50% at −1.9 V. A further increase in potential led to a decrease in efficiency due to hydrogen evolution occurring at high potentials. It also is important to note that CO was not produced in the N_2_-saturated electrolyte (Figure S2), indicating that the generated CO originated from the electrocatalytic reduction of CO_2_, rather than from ACN or MXene decomposition.

To ensure that TiO_2_/MXene was responsible for CO_2_RR and not its components, we compared the FE_CO_ of TiO_2_/MXene, MXene, and commercial TiO_2_ at −1.9 V. As shown in Figure 4D, the TiO_2_/MXene electrode exhibits a near 100% FE_CO_; however, almost no CO_2_ electroreduction resulted from electrodes fabricated using pure MXene or commercial TiO_2_. These results indicate the efficient CO_2_ reduction arises from the TiO_2_/MXene composite material. To confirm this hypothesis, the catalytic performance of the TiO_2_/MXene nanocomposite using different in situ growth times was studied (Figure S6). We found that increasing growth times up to 12 h produced higher FE_CO_ values, but excessive growth time reduced FE_CO_. We hypothesize that the observed decrease in FE_CO_ was due to poor conductivity caused by the excess synthesis of TiO_2_ on MXene, as confirmed by the electrochemical impedance spectroscopy (EIS) results in Figure S7. To demonstrate the stability of our TiO_2_/MXene system, CO_2_RR was performed at increasing timescales from 30 min to 3 h. As depicted in Figure S8, after 3 h of electrocatalysis, FE_CO_ only decreased by 13%, demonstrating certain stability for potential high-throughput applications. Overall, our results suggest that the in situ growth of TiO_2_ on MXene sheets produces efficient catalytic sites for CO_2_RR with high Faradaic efficiency and selectivity, which is an improvement compared to the other non-precious electrocatalyst materials reported, such as Bi nanoparticles [58], TiS_3_ [59], and TiO_2_ [60], as shown in Table S1.

**Figure 5:**
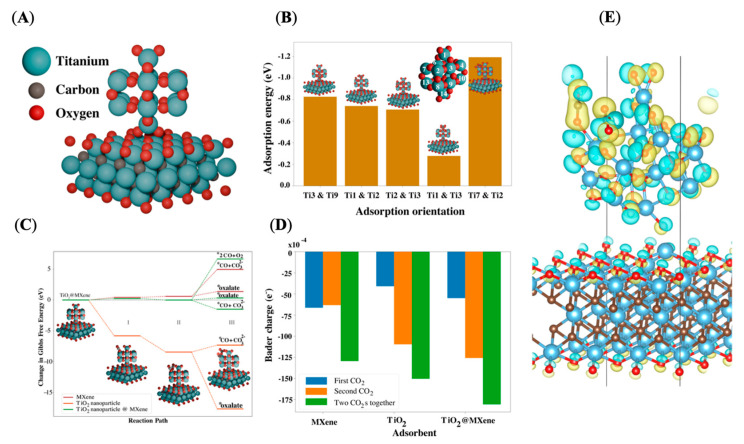
(**A**) The structure of the TiO_2_/MXene catalyst; (**B**) the adsorption energy of CO_2_ molecules above different titanium atoms of TiO_2_ nanoparticles on TiO_2_/MXene. (**C**) The energy profile of the reaction mechanism with MXene (brown), TiO_2_ (orange), and TiO_2_/MXene (green) as catalysts, with the end products of reaction paths noted above stage (III). The simulated intermediate structures of the TiO_2_/MXene system with the end products $CO^{*}$ and $CO_{3}^{2-}$ shown in the inset. (**D**) The charge density of adsorbed CO_2_ molecules on MXene, TiO_2_, and TiO_2_/MXene; (**E**) a side view of the Bader charge density illustration of adsorbed CO_2_ molecules on TiO_2_/MXene. Yellow and cyan denote electron accumulation and depletion.

To best understand the CO_2_RR mechanism on TiO_2_/MXene, density functional theory (DFT) calculations were performed using the Vienna ab initio simulation package with the Perdew–Burke–Ernzerh functional [61,62,63,64]. The projector-augmented wave pseudopotentials were implemented for the exchange correlation function and pseudopotential treatment [65,66]. The structure of the TiO_2_/MXene used in these considerations is shown in Figure 5A. The calculations were conducted on three systems: (1) TiO_2_ nanoparticles, (2) MXene, and (3) TiO_2_/MXene. The kinetic cutoff energy was 600 eV throughout the simulation. The Brillouin zone was sampled by a Monkhorst–Pack grid centered at the γ point with a k-point mesh of 3 × 3 × 3 for TiO_2_ and 3 × 3 × 1 for MXene and TiO_2_/MXene. All structures analyzed by DFT were fully optimized, and computations were continued until they met the convergence thresholds of having a total energy of 10^−6^ eV and atomic forces not exceeding 0.05 eV/Å. Other model details, e.g., the sizes of MXene (112 atoms) and TiO_2_ nanoparticles (42 atoms), the construction of slab models, and the nanoparticles (Figure S9), are included in the Supplementary Materials. The size of systems used for modeling was selected as a balance between computational efficiency and accuracy [61]. The analysis suggests that the (100) direction of TiO_2_ nanoparticles is most likely to be perpendicular to the (110) direction of MXene (Figure S10). Furthermore, the strong affinity between the TiO_2_ nanoparticles and MXene is primarily governed by the interaction involving oxygen atoms of TiO_2_ and titanium atoms of MXene, facilitated through oxygen dangling bonds. The resulting TiO_2_/MXene model is illustrated in Figure 5A and serves as the system upon which subsequent calculations are based.

To perform the adsorption energy analysis of CO_2_ onto the TiO_2_/MXene, we examined five geometrically unique adsorption configurations, as shown in Figure 5B, where the negative shift in adsorption energy indicates that the process is favorable. Interestingly, the adsorption free energy magnitude is comparatively weaker than that of other catalytic systems, placing TiO_2_/MXene within the optimal range for catalytic adsorption of CO_2_ [67,68]. From these results, we can ascertain that the CO_2_ molecules are adsorbed above the 17th and 12th Ti atoms, which are selected for further analysis. It is worth noting that the pattern of adsorption energy does not exist when CO_2_ is adsorbed onto MXene or TiO_2_ (Figures S11 and S12), where the elevated adsorption energy could potentially poison the adsorbent.

A Gibbs free energy change analysis was conducted to understand the energetics of intermediates while the reaction proceeds. The analysis was performed following three possible reaction paths for CO_2_ reduction in an aprotic environment. Step (R1) is the adsorption of the first CO_2_ molecule and reaction. Step (R2) is the successive adsorption of the second CO_2_ molecule onto the catalytic system. The reactions given in steps (R3a–c) represent the possible reductive paths of the two adsorbed CO_2_ molecules. Figure 5C depicts the Gibbs free energy changes for reactants and intermediates present in the reaction on TiO_2_/MXene, compared with pure MXene and TiO_2_ nanoparticles.

(R1) $$*+CO_{2}\to *CO_{2}$$

(R2) $$CO_{2}+*CO_{2}$$

(R3a) $$2*CO_{2}\to *CO+*O+*CO_{2}\to *CO+CO_{3}^{2-}$$

(R3b) $$2*CO_{2}+2e^{-}\to 2*CO_{2}^{2-}\to *oxalate(C_{2}O_{4})$$

(R3c) $$2*CO_{2}\to 2CO+O_{2}$$

Our free energy analysis leads to three unique systems for the potential catalysts, as shown in Figure 5C. (1) In the case of pure MXene (brown lines), we observe a gradual change in free energy as CO_2_ molecules are successively adsorbed. This incremental change suggests that reductive reactions on pure MXene surfaces are less likely to occur. (2) In the case of pure TiO_2_ nanoparticles (orange lines), the free energy change undergoes a significant negative shift upon adsorption of the first and second CO_2_ molecules. These adsorption processes lead to the formation of an oxalate group, accompanied by a substantial negative change in free energy (−9.20 eV). This oxalate group eventually dissociates into two CO_2_ molecules. (3) The case of the TiO_2_/MXene catalytic system is more nuanced. Initially, there is a slight positive change in free energy (0.07 eV) upon the adsorption of the first CO_2_ molecule, which reduces it to 0.02 eV when the second CO_2_ molecule is adsorbed near the first. In this scenario, three possibilities arise: (R3a) the two dimerized CO_2_ molecules can form one oxalate, (R3b) two CO molecules and one O_2_ molecule may be generated, and R3c) the formation of one CO molecule along with a carbonate anion (CO_3_^2−^) via intermediates such as ∗CO, ∗O, and activated CO_2_^2−^. Of these options, the first and second cases exhibit positive changes in free energy (0.06 eV and 6.64 eV, respectively), making these reactions less favorable. However, in the third scenario, the formation of one CO molecule and one CO_3_^2−^ shows a negative change in free energy (−0.51 eV), indicating that the CO_2_RR is energetically favorable in this direction. Further support for these findings comes from the Bader charge analysis shown in Figure 5D, where the net charge on adsorbed CO_2_ molecules reveals that they gain a higher negative charge when TiO_2_/MXene serves as a catalyst compared to pure MXene or TiO_2_. The results of the Bader analysis are visualized in Figure 5E, where the yellow shading indicates areas of charge accumulation, and cyan shows those of charge depletion. This suggests a significant charge transfer from TiO_2_/MXene to CO_2_ molecules. In the future, other product analyses may be performed including in situ spectroscopy [69], and possibly interfacial intermediate analyses through sum-frequency generation spectroscopy. Additionally, while our TiO_2_/MXene electrocatalyst showed high FE for the CO_2_RR to CO, it is at the cost of a high overpotential of 1.9 V vs. Ag/AgCl. Future strategies to reduce this overpotential include composite materials such as TiI_4_ to modify the electronic structure and promote more marketable reaction conditions [70].

## 3. Experiment

**Materials.** Ti_3_AlC_2_ (MAX) powders were purchased from Forsman (Beijing, China). Carbon paper (HCP020N, CP) was purchased from Hesen (Shanghai, China). Nickel foam (NF) was provided by Hefei Kejing Materials Technology (Hefei, Anhui, China). Nafion@117 solution was purchased from Sigma-Aldrich (St. Louis, MO, USA). Acetonitrile was purchased from Fisher Chemical (Pittsburgh, PA, USA). Ethanol and hydrochloric acid were purchased from Pharmco (Brookfield, CT, USA). Lithium fluoride was purchased from Alfa Aesar (Ward Hill, MA, USA). Potassium hexafluorophosphate was purchased from Oakwood Chemical (Estill, SC, USA). Deionized (DI) (18.2 MΩ cm) water was produced by using a Milli-Q ultrapure system (Thermo Scientific Barnstead E-Pure, Waltham, MA, USA).

**Preparation of MXene.** Ti_3_C_2_T*_x_* MXene was obtained via an HF etching method [71]. A total of 100 mg of LiF was added into 2 mL of 9 M HCl solution in an ice bath before 100 mg of MAX was added into the solution. After etching at 35 °C for 24 h, the obtained MXene was washed 8 times with DI water and centrifuged at 3000 rpm for 1 h. The collected MXene was freeze-dried and stored in a vacuum desiccator.

**Preparation of TiO_2_/MXene.** As illustrated in Figure 1, 20 mg of Ti_3_C_2_T*_x_* MXene was dispersed in 2 mL ethanol and sonicated at 400 W for 2 h. The suspension was transferred into a 40 mL PTFE autoclave for solvothermal treatment at 120 °C for 12 h. Afterward, the as-prepared suspension was centrifuged at 3000 rpm for 20 min. The obtained solid was freeze-dried and stored in a vacuum desiccator.

**Characterizations.** X-ray diffraction (XRD) patterns were analyzed by using a Rigaku Miniflex II X-ray diffractometer (Rigaku Americas, The Woodlands, TX, USA) with Cu Kα radiation (λ = 1.5406 Å). Transmission electron microscopy (TEM), high-resolution transmission electron microscopy (HR-TEM) images, and elemental mapping analysis were collected using a FEI Tecnai G2 F20 FE-TEM (FEI, Hillsboro, OR, USA) microscopy system. X-ray photoelectron spectroscopy (XPS) measurements were performed using a Kratos Axis Ultra instrument (Kratos Analytical Ltd., Manchester, UK).

**Preparation of the Working Electrode.** A total of 4 mg of th TiO_2_/MXene catalyst was suspended in a mixture of 960 μL Di-water and 40 μL Nafion@117 solution, followed by a 2 h sonication to form a homogeneous ink. A total of 100 μL of the ink (0.4 mg of catalyst) was deposited onto a carbon paper strip with an area of 1 cm^2^ and dried in a vacuum desiccator.

**Electrocatalytic CO_2_ Reduction.** All potentials are reported with respect to the Ag/AgCl reference electrode. Electrochemical CO_2_ reduction was performed using a Gamry Interface 1000 electrochemical workstation. All electrochemical measurements were conducted in a three-electrode gas-sealed single-compartment glass cell, as shown in Figure S1. TiO_2_/MXene supported on CP (0.4 mg/cm^2^) was used as the working electrode while Ag/AgCl (0.1 M KPF_6_ in acetonitrile) and nickel foam (NF) (2 × 1 cm) electrodes were used as the reference and counter electrodes, respectively, and conducted in a 0.1 M KPF_6_ acetonitrile solution. The electrolyte solution was purged with CO_2_ for 30 min before measurement. Cyclic voltammogram (CV) scans were performed at 10 mV/s with IR compensation. Electrochemical impedance spectroscopy (EIS) analysis was performed at −1.9 V vs Ag/AgCl in the frequency range of 10^4^–10^−2^ Hz. Controlled potential coulometry was performed at −1.9 V for 30 min. Gas products produced from electrolysis were measured by an SRI 8610C (SRI Instruments, Torrance, CA, USA) gas chromatograph equipped with a thermal conductivity detector (TCD) and a flame ionization detector (FID), using argon as the carrier gas. Faradaic efficiency (FE) for CO was calculated by

(1) $$\varepsilon =\frac{e\times F\times M}{Q}\times 100\%,$$

where *ε* is Faradaic efficiency, F=96485 C/mol is Faraday’s constant, e=2 is the number of transferred electrons for CO_2_RR, *M* is the amount of CO (mol) and is obtained from the corresponding peak area of the FID curve from GC experiments, and *Q* is the charge obtained from the reaction.

## 4. Conclusions

In this work, we demonstrated the catalytic ability of novel TiO_2_/MXene nanocomposite electrodes for an efficient electrochemical CO_2_RR. The nanocomposite TiO_2_/MXene electrodes were easily fabricated using cost-effective solvothermal and drop-cast techniques. Electrochemical characterization of the electrode through cyclic voltammetry showed that it behaved as a capacitor in the absence of CO_2_ but a cathodic current in its presence indicated a reduction reaction at the electrode. The electrochemical performance of the CO_2_RR to produce CO showed an excellent FE of 99.41% at −1.9 V (vs. Ag/AgCl), by far outperforming the individual components. A DFT-level analysis of the CO_2_RR on TiO_2_/MXene electrodes revealed the likely mechanism producing CO and a carbonate anion through ∗CO, ∗O, and activated CO_2_^2−^ intermediates, as well as two less likely pathways which resulted in an oxalate or two CO molecules with one O_2_. Subsequent Bader charge analysis showed that TiO_2_/MXene behaves as a catalyst in the CO_2_RR compared to its TiO_2_ or MXene components alone. Additional work is still needed to confirm the proposed reaction mechanism by quantifying reaction products through mass-spectrometry and monitoring intermediate formation through sum-frequency generation spectroscopy. In sum, easy-to-produce TiO_2_/MXene nanocomposite electrodes may hold great utility in the harvesting and use of atmospheric CO_2_ through an efficient CO_2_RR to combat the pressing global climate and energy crises.

## Data Availability

Data will be provided by the corresponding author upon reasonable request.

## Supplementary Materials

The following supporting information can be downloaded at: https://www.mdpi.com/article/10.3390/molecules30194025/s1, Figure S1: Schematic of the CO_2_ reduction reaction electro-reduction cell; Figure S2. High-resolution XPS spectrum of F1s for TiO_2_/MXene; Figure S3. High-resolution XPS spectrum of F1s for TiO_2_/MXene; Figure S4. EDX result of TiO_2_/MXene; Figure S5. Faradaic efficiencies (FE) for CO by TiO_2_/MXene when electrolyte is saturated with CO_2_ (red) or N_2_ (green); Figure S6. Faradaic efficiencies (FE) for CO production by TiO_2_/MXene at different TiO_2_ growth times; Figure S7. The EIS curves of MXene, TiO_2_/MXene at solvothermal time of 1, 4, 12, and 36 h; Figure S8. Faradaic efficiencies (FE) for CO production by TiO_2_/MXene at different electrolysis time; Figure S9. (A) Surface energy of specified surfaces with miller indices of TiO_2_ and (B) the TiO_2_ nanoparticle constructed using Wulff construction method; Figure S10. Adsorption energy of TiO_2_ nanoparticle with various geometric orientations above MXene; Figure S11. Adsorption energy of two CO_2_ molecules on MXene at symmetrically distinct positions; Figure S12. Adsorption energy of a CO_2_ molecule on TiO_2_ at symmetrically distinct positions. Table S1. Comparison of electrocatalytic CO_2_RR activities of various nonprecious catalysts with those reported in the literature.
