# Supplementary material for: Interface Catalysts of In Situ-Grown TiO2/MXenes for High-Faraday-Efficiency CO2 Reduction
Source: Molecules. 2025 Oct 9;30(19):4025. doi: 10.3390/molecules30194025 (PMC12526155; doi:10.3390/molecules30194025)
Supplement: Supplementary file 1 [file molecules-30-04025-s001.zip › molecules-3843972-supplementary.pdf]

Supporting Information

Interface Catalysts of *in-situ* Growth TiO_2/MXene for Near 100% Faraday Efficiency CO_2 Reduction

Shaun Debow[1#], Zichen Shen[2#], Arjun Sathyan Kulathuvayal[3#], Fuzhan Song[2#], Tong Zhang[2], Haley Fisher[2], Jesse B. Brown[2], Yuqin Qian[2], Zhi-Chao Huang-Fu[2], Zachary Zander, Mark S. Mirotznik [4], Robert L. Opila[5], Yanqin Su[3], and Yi Rao[2*]

[1]U.S. Army Combat Capabilities Development Command Chemical Biological Center, Research & Operations Directorate, Aberdeen Proving Ground, MD 21010, USA
[2]Department of Chemistry and Biochemistry, Utah State University, Logan, Utah 84322, USA
[33]School of Aerospace and Mechanical Engineering, University of Oklahoma, Norman, OK, 73019, USA
[4]Department of Electrical and Computer Engineering, University of Delaware, Newark, DE, 19711, USA
[5]Department of Materials Science and Engineering, University of Delaware, Newark, DE, 19711, USA

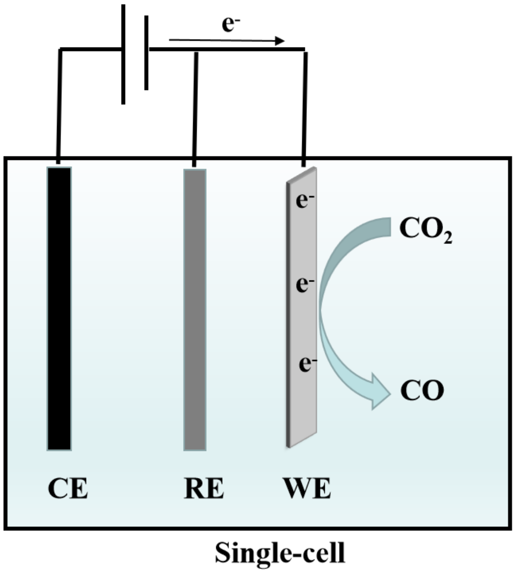

Figure S1. Schematic of the CO_2 reduction reaction electro-reduction cell. WE: working electrode (0.4 mg of TiO_2/MXene on 1 cm^2 carbon paper). RE: reference electrode (Ag/AgCl electrode). CE: counter electrode (Ni foam). Electrolyte: acetonitrile solution with 1 M KPF_6.

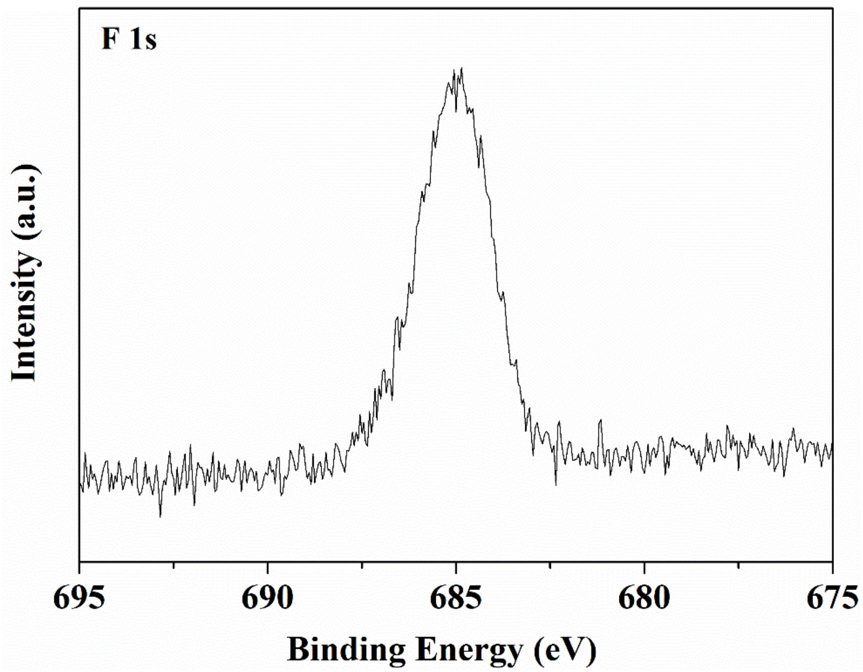

Figure S2. High-resolution XPS spectrum of F1s for TiO_2/MXene.

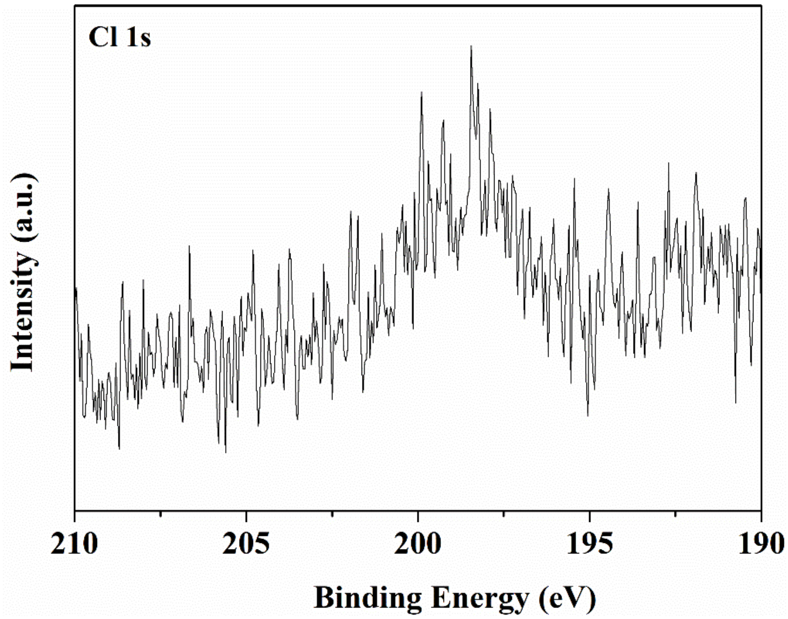

Figure S3. High-resolution XPS spectrum of F1s for TiO_2/MXene.

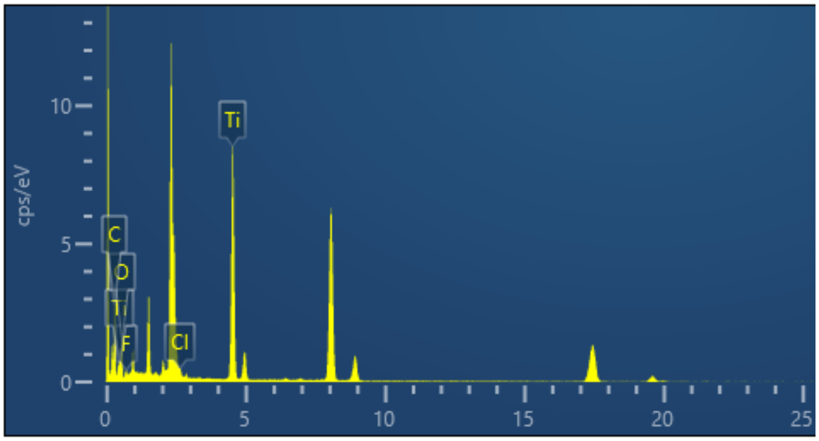

Figure S4. EDX result of TiO_2/MXene.

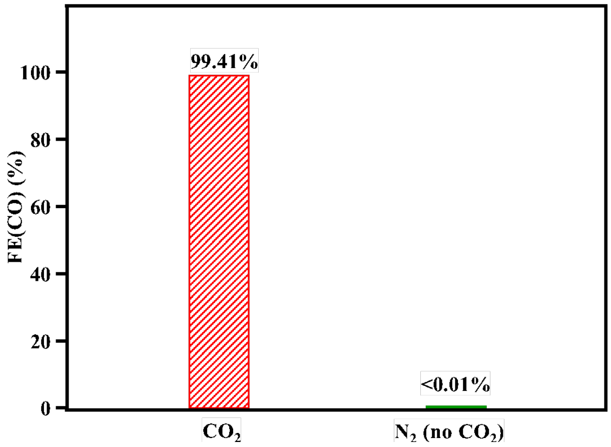

Figure S5. Faradaic efficiencies (FE) for CO by TiO_2/MXene when electrolyte is struated with CO_2 (red) or N_2 (green).

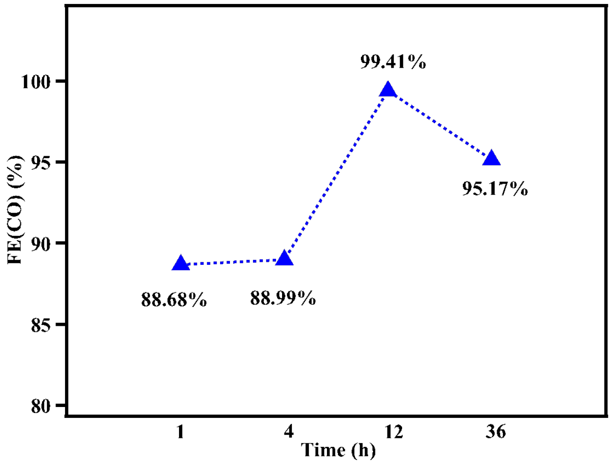

Figure S6. Faradaic efficiencies (FE) for CO production by TiO_2/MXene at different TiO_2 growth times.

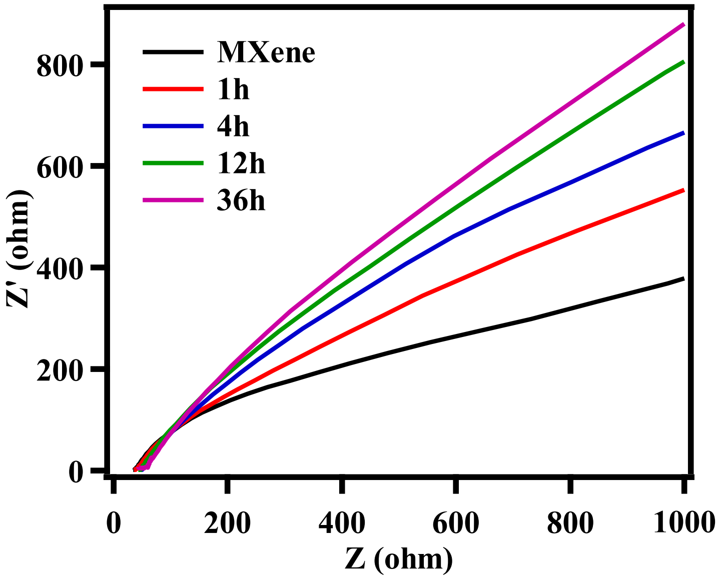

Figure S7. The EIS curves of MXene, TiO_2/MXene at solvothermal time of 1, 4, 12, and 36 h.

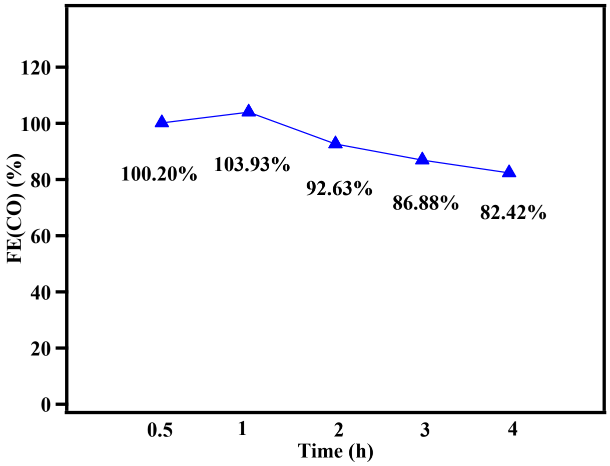

Figure S8. Faradaic efficiencies (FE) for CO production by TiO_2/MXene at different electrolysis time.

Table S1 Comparison of electrocatalytic CO2RR activities of various nonprecious catalysts with those reported in the literature.

Catalyst	Potential	Faradaic efficiency	Stability	Reference
TiO_2/MXene	-1.9 V vs. Ag/AgCl	99.41 %	3 h	This work
Bi-ED	-2.0 V vs. Ag/AgCl	96.1 %	2 h	ACS Catal. 2016, 6, 6255−6264
TiS_2	-0.5 V vs. RHE	83 %	16 h	ACS Catal. 2020, 10, 66−72
FePEGP	-2.2 V vs. Fc/Fc+	98 %	/	Inorg. Chem. 2021, 60, 3843−3850
O-EGaIn	-2.4 V vs. RHE	86 %	10 h	Appl. Surf. Sci. 2022, 612, 155934
Cu/CuxO.GO	-0.28V vs. NHE	40 %	/	J CO2 UTIL. 2020, 39, 101178
d-Mo2C	-2.24 V vs. SCE	90 %	1 h	Chem. Commun., 2021, 57, 1675
Ag	−1.9 vs. Fc/Fc+	~100 %	/	Sci. China Chem. 2025, 68, 2110−2116

TiO_2 Nanoparticle Modeling

To determine the predominant surfaces of TiO_2 nanoparticles, the surface energies of all identified surfaces from the XRD analysis are included in the density functional theory (DFT)-based surface energy analysis (Figure S9(A)). Surface energy is calculated using Equation S1:

$$\gamma = \frac{E_{slab} - NE_{bulk}}{2A}, \tag{S1}$$

where E_{slab} is the energy of the slab supercell, N is the number of atoms in the slab, E_{bulk} is the energy per atom in the bulk supercell, and A is the area of the surface under investigation in the slab model. Here, dividing by 2 accounts for the two identical surfaces exposed to the vacuum.

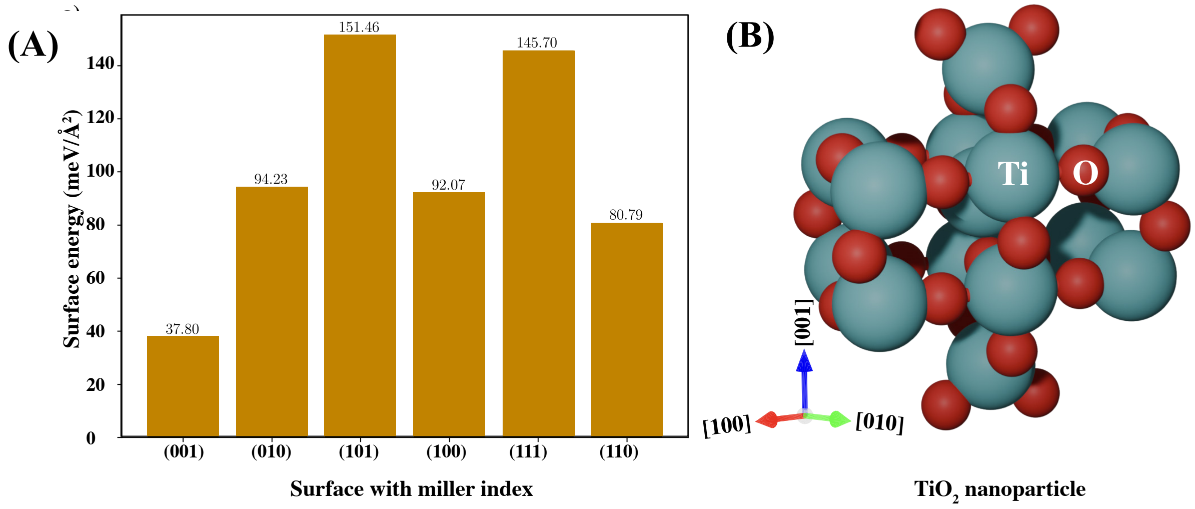

Figure S9. (A) Surface energy of specified surfaces with miller indices of TiO_2 and (B) the TiO_2 nanoparticle constructed using Wulff construction method.

This study employed Wulff construction to model the TiO2 nanoparticle (Wulff, G. *Zeitschrift für Kristallographie - Crystalline Materials* **1901**, 34, 449–530.). In this approach, a polyhedron of TiO_2 is constructed (Figure S9(B)) using the planes ((001), (100), and (110)) that have the lowest surface energy compared to other planes (Mathew, K. et al., *Comput. Mater. Sci.* **2016**, 122, 183–190.). We can reasonably assume that faces having relatively low surface energy will be the most prevalent equilibrium shape of the generated nanoparticle (Barmparis, G. D et al., *Beilstein J. Nanotechnol.* **2015**, 6, 361–368.). Considering the computational demand, the average radius of TiO_2 nanoparticles was limited to 0.50 nm, encompassing 42 atoms and forming a nanoparticle with 14 formula units of TiO_2. Although smaller than typical experimental sizes, this chosen TiO_2 nanoparticle size is feasible for DFT calculations, given the constraints in handling larger systems. Importantly, it is considered adequately large compared to the adsorbent – the MXene with oxygen as surface terminations ($Ti_3C_2O_2$. This helps to avoid a strong binding effect caused by highly undercoordinated atoms in TiO_2 nanoparticles which could lead to unrealistically high reactivity and overestimation of overpotentials, as Lim and Wilcox previously explored in their study on the oxygen reduction reaction (Lim and Wilcox *J. Phys. Chem. C* **2012**, 116, 3653–3660.).

Adsorption energy

The adsorption energy calculations mentioned in this study are performed using Equation S2;

$$E_{ads} = E_{adsorbent+adsorbate} - E_{adsorbent} - E_{adsorbate}, \quad \text{(S2)}$$

where E_{ads} represents the adsorption energy and $E_{adsorbent+adsorbate}$, $E_{adsorbent}$, and $E_{adsorbate}$ denote the DFT energies associated with the system comprising both adsorbent and adsorbate, the bare adsorbent, and the isolated adsorbate, respectively.

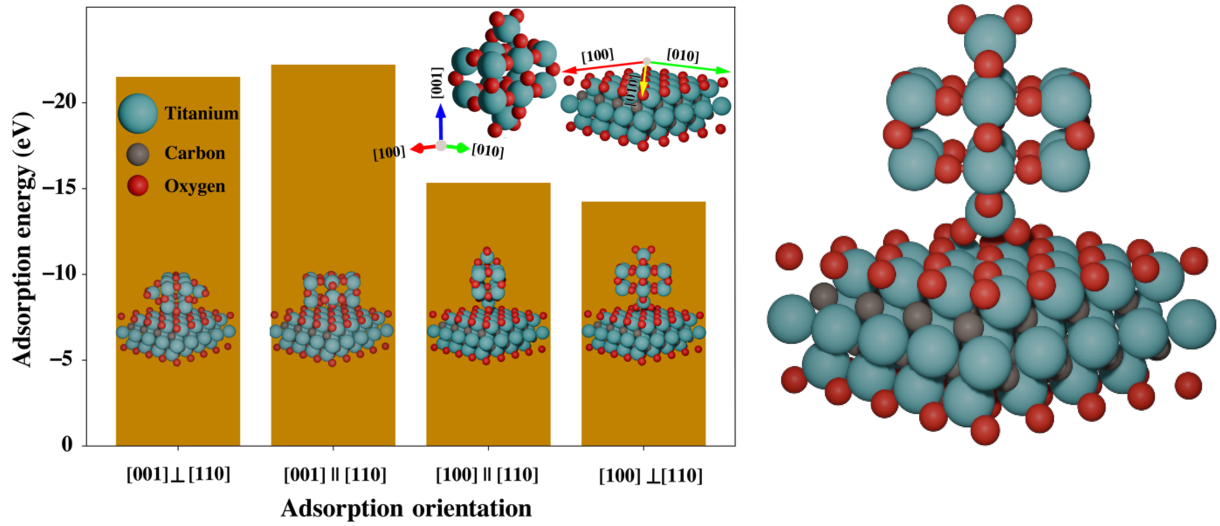

Figure S10. Adsorption energy of TiO_2 nanoparticle with various geometric orientations above MXene.

Free Energy Analysis

The free energy change of intermediates was calculated using Equation S3:

$$\Delta G_{ads} = E_{ads} + \Delta E_{ZPE} - T\Delta S, \quad \text{(S3)}$$

where $T\Delta S$ is the corrected entropic change and ΔE_{ZPE} is the difference in zero-point energy derived from the vibrational frequencies.

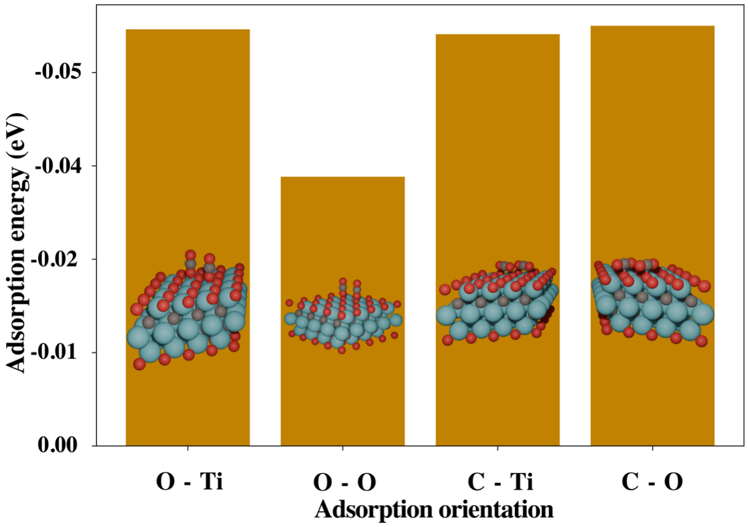

Figure S11. Adsorption energy of two CO_2 molecules on MXene at symmetrically distinct positions. For the *x*-axis, the labels indicate which element of CO_2 is bound to which element of MXene. The illustrations in each bar represent the spatial geometry of CO_2 molecules during adsorption.

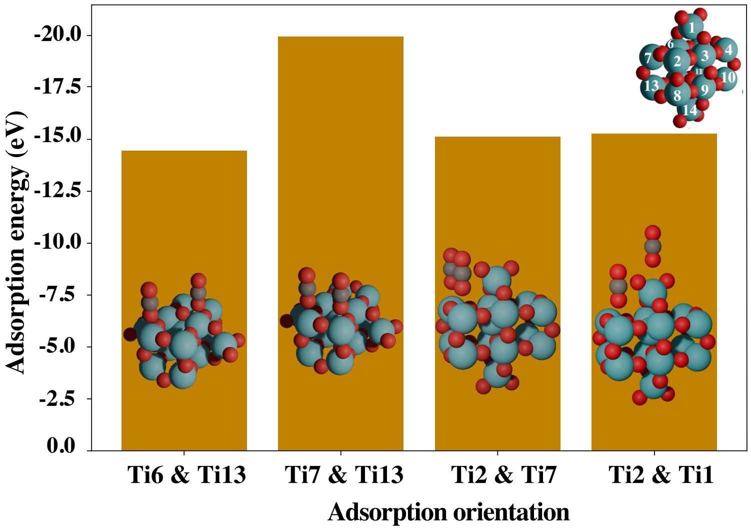

Figure S12. Adsorption energy of CO_2 molecule on TiO_2 at symmetrically distinct positions. The index of titanium atoms above which adsorption takes place is given in the *x*-axis labels. Indexing is defined in the inset.
